# Ninety Day Toxicity and Toxicokinetics of Fluorochloridone after Oral Administration in Rats

**DOI:** 10.3390/ijerph120504942

**Published:** 2015-05-06

**Authors:** Suhui Zhang, Xiaoqin Cheng, Yu Wang, Junpei Fan, Rui Li, Su Zhou, Shihong Liu, Jingmin Shi, Jie Sun, Yue Hu, Chaojin Xu, Chunhua Wu, Xiuli Chang, Liming Tang, Zhijun Zhou

**Affiliations:** 1 School of Public Health, Fudan University, Shanghai 200032, China; E-Mails: 12111020016@fudan.edu.cn (S.Z.); 13211020060@fudan.edu.cn (S.L.); chwu@shmu.edu.cn (C.W.); xlchang@shmu.edu.cn (X.C.); 2 Pharmacology and Toxicology Department, Shanghai Institute for Food and Drug Control, Shanghai 201203, China; E-Mails: cxq19850429@aliyun.com (X.C.); ohyue@163.com (Y.W.); 13761243089@126.com (J.F.); lirui200901@163.com (R.L.); hello-world-zhou@163.com (S.Z.); jingmin.shi@nih.gov (J. Shi); sjabcd1983@163.com (J. Sun); huyue86816@hotmail.com (Y.H.); ratxu1972@126.com (C.X.); tangliming@smda.gov.cn (L.T.)

**Keywords:** fluorochloridone, ninety days toxicity, gavage, toxicokinetics, UPLC-MS/MS, rat

## Abstract

The ninety day toxicity and toxicokinetics of fluorochloridone (FLC) were accessed in Wistar rats. Animals were gavaged with FLC at doses of 31.25 mg/kg, 125 mg/kg and 500 mg/kg for ninety days, followed by thirty days for recovery. On the 1st, 60th, 75th and 90th days of the dosing phase, plasma of ten animals of all groups treated with FLC was collected for toxicokinetic analysis of FLC by an UPLC-MS/MS method. Numerous changes in body weight, hematology, serum chemistry, and organ weight ratios were observed by the 45th and 90th dosing day. Most changes in groups treated with FLC were absent on the last recovery day. Testis and epididymis lesions were consistently seen in histopathological observations on the 45th, 90th dosing day and the last recovery day. Repeated administration of FLC increased the level of testosterone in serum in male rats on the 90th dosing day. FLC plasma concentrations could be detected in all animal drug-treated groups during the dosing phase, and a dose proportional relationship was seen between FLC dose and AUC or C_max_. This study will support future studies on the mechanism of FLC-induced toxicity.

## 1. Introduction

Among the three major classes of pesticides global herbicide sales have been in first place in recent years at around 48% of the total. A large number of herbicides have been developed by the agrochemical industry and are widely used around the world. The proportion of herbicide users in various areas is different and the differences are becoming bigger and bigger in recent years. Economically developed regions such as Europe and North America are the largest consumers of herbicides. Most herbicides exhibit low acute toxicity, and there are very few reports of acute poisoning incidents, but herbicide residues in vegetables and other foods can result in chronic harmful effects to humans and animals, and it is noted that chronic hazards, such as nervous system damage [1,2,3], hormone imbalance [4], fertility disorders [5], immune system dysfunction [6], and so on, pose a threat to human health.

The herbicide fluorochloridone (FLC) is widely used for pre-emergence control of broad-leaved weeds and annual grasses in carrot, sunflower, potato and several other crops. According to the European Food Safety Authority [7], there is no evidence suggesting that fluorochloridone is genotoxic, carcinogenic or neurotoxic. The target organs of FLC as a potential endocrine disruptor in male rats are the testis and epididymides [7], but there is no data to confirm this. In the report, the heart, major vessels and the haematopoietic system were also identified as potential target organs of FLC in rat [7]. Acute and short-term NOAELs were set from these critical studies at 20 mg/kg bw/day, where the LOAEL is 25 mg/kg bw/day [7]. In a recent investigation, we discovered that oral administration of FLC (30 mg/kg·bw/day–750 mg/kg·bw/day) for 28 days could damage the testis of adult Sprague Dawley (SD) rats by inducting oxidative stress [8]. Abnormal cell-cycle progression, cellular mitodepressive activity and chromosomal abnormalities induced by FLC in *Allium cepa* root meristematic cells were also demonstrated [9]. The genotoxic and cytotoxic effects of pure FLC and its two main formulations in Chinese Hamster Ovary K1 (CHO-K1) cells using several end-points were reported [10]. In addition, FLC and its two commercial formulations could induce single-strand DNA breaks *in vitro* in mammalian cells [11]. GC-MS methods of analysis are recommended for residues of FLC in plants, soil, water and air, but there is a data gap for a suitable method of analysis for body fluids and tissues [7].

Based on the European Food Safety Authority (EFSA) report, there are no more detailed technical data about all the target organs or potential target organs in rats. Consistency of toxicity assessment data on FLC derived from different institutions is also important for revealing the characteristics of FLC toxicity. In this study, we report the 90 day toxicity and toxicokinetics (TK) of FLC in Wistar rats to characterize more detailed findings about target organs. Rats were gavaged with FLC daily for 90 days, followed by a 30 day recovery phase. The following clinical signs were observed: changes in body weight, food consumption, serum biochemistry, hematology, hormone level in serum, macroscopic findings at necropsy and histopathologic alterations. In addition, the TK profiles of FLC were reported using a validated UPLC-MS/MS assay by comparing different doses of FLC on the 1st, 60th, 75th and last (90th) dosing day.

## 2. Materials and Methods

### 2.1. Materials

Fluorochloridone (purity > 95.5%) was purchased from Jiangxi Anlida Chemical Co., Ltd. (Jiangxi, China). A FLC standard (purity 99.0%) was purchased from Sigma-Aldrich (Seelze, Germany). Verapamil was provided by the Shanghai Institute for Food and Drug Control (Shanghai, China) as an internal standard for the UHPLC-MS/MS analysis of FLC. Mass spectroscopy grade acetonitrile, methanol, and formic acid were purchased from Merck (Darmstadt, Germany). All other chemicals used were of the highest commercial grade available.

### 2.2. Experimental Animals and Housing Conditions

This study was conducted at the Shanghai Institute for Food and Drug Control (SIFDC, Shanghai, China) and was carried out according to the OECD Guidelines for the “Repeated Dose 90-day Oral Toxicity Study in Rodents” [12]. All protocols were approved by the Institutional Animal Care and Use Committee of SIFDC. Six weeks male and female Wistar rats were obtained from Shanghai SLAC Laboratory Animal Co., Ltd. (Shanghai, China). Animals were kept in a room maintained at 23 ± 2 °C, relative humidity of 40%–70%, under a 12 h light/dark cycle.

### 2.3. Sample Preparation Procedures

FLC was suspended in 0.5% (w/v) sodium carboxymethyl cellulose (CMC-Na) used as a vehicle at concentrations of 1.56 mg/mL, 6.25 mg/mL and 25 mg/mL and fresh samples were prepared once every three days. The suspension was stirring during oral administration at room temperature.

### 2.4. Experimental Design

One hundred and ninety Wistar rats were fed a standard diet for 10 days to adapt to the environment before the experiments, and then divided into four groups at random by body weight. Group 0 was the control group which was gavaged with 0.5% (w/v) CMC-Na (*n* = 40, G0). Group 1 was gavaged with a dose of 31.25 mg/kg FLC (*n* = 50, G1). Group 2 was gavaged with a dose of 125 mg/kg FLC (*n* = 50, G2). Group 3 was gavaged a dose of 500 mg/kg FLC (*n* = 50, G3). Each group included half male and half female rats. Forty rats of each FLC group (G1, 2 and 3) and control group (G0) used in the ninety day toxicity test were gavaged with FLC for 90 days (Weeks 1–13) and feed without FLC to recover for 30 days (Weeks 14–17). The remaining ten animals of each FLC group (G1, G2 and G3) used in the TK test were gavaged with FLC for 90 days without a recovery phase. Body weights of all animals were measured once a week and the volume of FLC gavaged to each animal was adjusted according to their body weights.

On the 46th, 91th and 121th experimental date during the 90 day dosing phase and 30 day recovery phase of the 90 day toxicity test, 12, 16 and 12 animals, respectively, including half male and female rats, were fasted 18 h but with free access to water in a metabolic cage before histopathological examination. Animals’ blood were drawn via the abdominal aorta and urine of each animal was accumulated for clinical biochemistry, hematology, coagulation, hormone detection and routine urine tests. The main organs like brain, heart, liver, lung, kidney, adrenal gland ratio, thymus, spleen, testis, epididymis, prostate, seminal vesicle, ovary, uterus and fallopian tube were removed and weighed immediately for histopathology. Other organs and tissues like the eyeballs, lymphonodus, salivary glands, pancreas, stomach, intestine, bladder, vagina, sciatic nerve, pituitary gland, spine, and so on were also removed for histopathology. On the 1st, 60th, 75th and 90th experimental days of the TK test, G1, G2 and G3 included eight time points at 30 min. and 1, 2, 3, 5, 8, 10 and 24 h after administration before the next dosing on each experimental date. Blood samples of ten animals of each group were collected (anticoagulant: heparin sodium) via the orbital sinus.

### 2.5. Clinical Observations

Clinical observations were performed every day for all animals prior to the oral administration and during the course of gavage. Body weight data and food consumption data were collected once a week during the study period.

### 2.6. Clinical Pathology

Serum and plasma of all animals was accumulated by centrifugation at 804× g for 10 min. at 4 °C (Hettich Rotanta 460R Centrifuge, Tuttlingen, Germany). Biochemistry of the serum samples was tested using a Hitachi 7060 Automatic Biochemical Analyzer (Naka, Japan). Hematology of plasma samples (with K_2_-EDTA added as anticoagulant) was analyzed on a Bayer ADVIA 120 Automatic Blood Cell Analyzer (Leverkusen, Germany). Plasma samples (with added sodium citrate as anticoagulant) were tested for coagulation function on a Sysmex CA1500 Full-Automatic Coagulation Analyzer (Kobe, Japan). Urine samples were collected for routine urine tests (Bayer HealthCare Clinitek Status Urine Analyzer, NY, USA). The levels of testosterone (T), estradiol (E2), follicle-stimulating hormone (FSH) and luteinizing hormone (LH) in serum were measured by radioimmunoassay.

Blood samples were collected from the abdominal aorta on the 46th, 91th and 121th experimental date for clinical biochemistry, hematology, coagulation and routine urine tests. The following indicators of serum were determined: alanine aminotransferase, alkaline phosphatase, aspartate aminotransferase, γ-glutamyltraspeptidase, total protein, albumin, creatine phosphokinase, glucose, cholesterol, triglycerides, total bilirubin, urea nitrogen, creatinine, uric acid, glycosylated hemoglobin, high density lipoprotein, low density lipoprotein, sodium ion, potassium ion, chloride ion. Samples were collected in tubes containing K_2_-EDTA for the hematology analyses and the following indicators were determined: total leukocyte count, differential leukocyte count, hemoglobin, red blood cell count, hematocrit, red blood cell distribution width—coefficient of variation, mean corpuscular hemoglobin concentration, platelet count, absolute reticulocytes count and so on. Samples collected in tubes containing sodium citrate were analyzed for coagulation function and the following indicators were determined: activated partial thromboplastin time, thromboplastin time, fibrinogen, fibrinogen concentration, prothrombin time, prothrombin time international normalized ratio. Urine samples were tested for pH, urobilinogen, glucose, *etc.*

### 2.7. Necropsy and Histopathology

**Figure 1:**
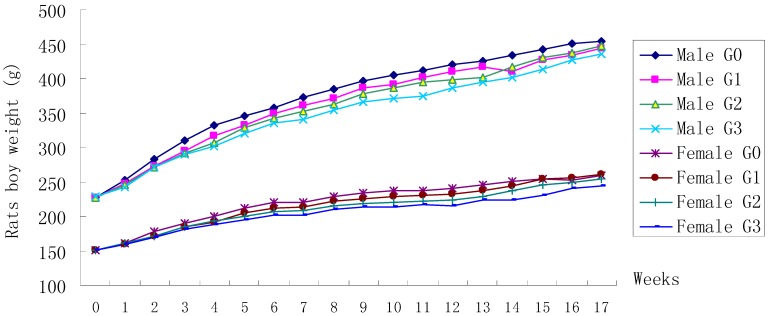
Mean body weight trend chart of Wistar rats treated with FLC. G0 means Group 0 (Control, 0.5% CMC-Na), G1 means Group 1 (FLC 31.25 mg/kg), G2 means Group 2 (FLC 125 mg/kg), G3 means Group 3 (FLC 500 mg/kg).

**Figure 2:**
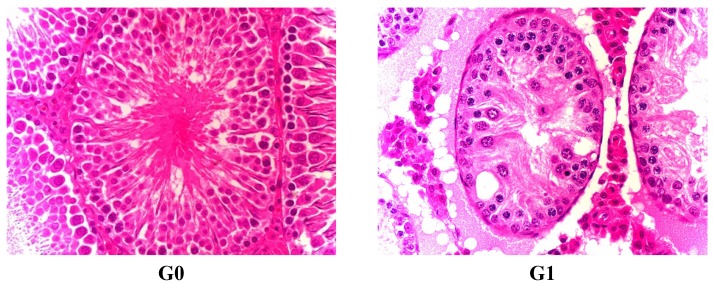

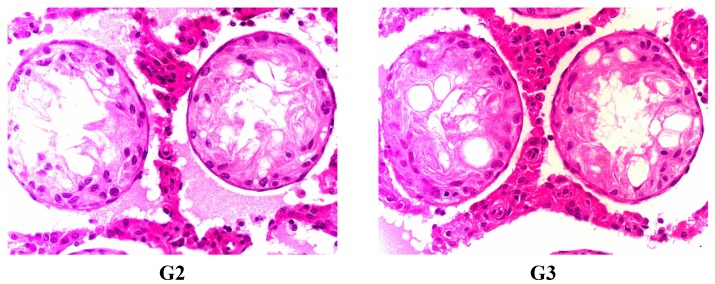
Lesion of testis in male rats treated with FLC on day 45 for atrophic and degenerating tubules, stained with H&E. G0 means Group 0 (0.5% CMC-Na), G1 means Group 1 (FLC 31.25 mg/kg), G2 means Group 2 (FLC 125 mg/kg), G3 means Group 3 (FLC 500 mg/kg).

Animals were sacrificed by exsanguination while under deep anesthesia. The final body weight was recorded, and a complete gross necropsy was conducted. Major organs were weighed, and a complete standard set of tissues (>30 per animal) were preserved in neutral buffered formalin and male reproductive organs were preserved in Davison’s fixative. Some main organs like brain, heart, liver, lung, kidney, adrenal gland ratio, thymus, spleen, testis, epididymis, prostate, seminal vesicle, ovary, uterus and fallopian tubes were weighted immediately. Main organ coefficients (organ weight × 100 / body weight) were calculated. Organs and tissues were embedded in paraffin, sectioned, stained with hematoxylin and eosin (H&E), and examined by histological analysis (Figure 1 and Figure 2). Histological diagnosis was performed according to [13,14].

### 2.8. Plasma FLC Determination

Plasma was cumulated by centrifugation at 804 × g for 10 min. at 4 °C (Hettich Rotanta 460R Centrifuge) and the supernatant was stored at −70 °C until analysis. Plasma samples were analyzed for FLC by an UPLC-MS/MS method. Briefly, a 100 μL aliquot of plasma was transferred into a 1.5-mL tapered plastic centrifuge tube, followed by the addition of 10 μL of verapamil (0.05 μg/mL) as an internal standard. The mixture was mixed for 30 s, followed by an addition of 300 μL of acetonitrile. The mixture was vortexed for 3 min and centrifuged at 36,670 × g for 10 min at 4 °C (Sartorius SIGMA 3K30 Centrifuge, Göttingen, Germany). An aliquot of the upper phase (1 μL) was subsequently injected into the UPLC-MS/MS system for analysis. An API 5000 triple quadrupole mass spectrometer (AB SCIEX, Boston, MA, USA) accompanied by Ultra Performance Liquid Chromatography was performed on an ACQUITY UPLC BEH C_18_ column (2.1 mm × 50 mm, 1.7 μm, Waters, Milford, MA, USA). The mobile phase was 0.1% formic acid in water-0.1% formic acid in acetonitrile (50:50, v/v) at a flow rate of 0.6 mL/min. The ion-spray potential was set at 5.5 kV, and the source temperature was 450 °C. Multiple reaction monitoring (MRM) were performed by using nitrogen as the collision gas. The analysis were detected by monitoring the transtitions *m/z* 312.0 → *m/*z 292.0 for FLC and *m/z* 456.2 → *m/z* 165.2 for verapamil (IS), with collision energies (CE) of 31 eV and 41 eV, respectively. The declustering potentials (DP) of FLC and verapamil were 188 V and 218 V. The calibration equation was determined by least-squares linear regression (weighted 1/x × x) over the range 3–3000 ng/mL in plasma.

### 2.9. Statistical Analysis

All measurements are expressed as the means ± standard deviation. Statistical analysis was performed with one-way analysis of variance (ANOVA) and Student’s *t* test using SPSS 19.0. *p*-values lower than 0.05 were considered to be significant. Toxicokinetic parameters (T_max_, C_max_, Clearance [CLz/F] and area under the curve [AUC]) in rats were assessed by a non-compartmental method using the Drug and Statistics 2.1 (DAS 2.1) software package (Mathematical Pharmacology Professional Committee of China, Shanghai, China).

## 3. Results

### 3.1. In-Vivo Observations

No animals died during dosing phase and there were no abnormal clinical signs indicating of an adverse effect arising from the oral administration of FLC. A difference in the body weight was observed intermittently in male animals treated by FLC 125 mg/kg (Weeks 2–4 and 7–13) and male animals treated by FLC 500 mg/kg (Weeks 1 and 3–13) with comparison to control-treated male animals (Figure 1). A difference in the body weight was observed intermittently in female animals treated by FLC 125 mg/kg (Weeks 5–7 and 13–15) and female animals treated by FLC 500 mg/kg (Weeks 3–7 and 13–15) compared to control-treated female animals (Figure 1). The difference in the body weight was not significant in male and female animals treated with FLC 125 mg/kg and 500 mg/kg during the last two recovery weeks compared to control-treated animals. Mean food consumption data indicated no treatment-related changes during the dosing phase or the recovery phase.

### 3.2. Clinical Pathology

Serum chemistry parameters are shown in Table 1 and Table 2. A decreased value of alkaline phosphatase, aspartate aminotransferase and alanine aminotransferase were observed in both male and female FLC-treated rats of all groups compared to the control-treated rats on the 45th dosing day and the last dosing day. A decreased level of aspartate aminotransferase and alanine aminotransferase were observed in the male and female FLC-treated rats of G3 compared to the control-treated rats on the last dosing day, respectively. An increased value of cholesterol was observed on both male and female FLC-treated rats of G2 and G3 compared with the control-treated rats on the last dosing day. An increased value of glucose was observed in the female FLC-treated rats of G1 compared to the control-treated rats on the 45th dosing day. On the last dosing day, glycosylated hemoglobin was tested to predict the influence of FLC on the pancreas. An obviously decreased value of glycosylated hemoglobin was observed in both male and female FLC-treated rats of G2 and G3 compared to the control-treated rats. A moderately increased value of total protein, albumin, total bilirubin and urine nitrogen were observed in the male and female FLC-treated rats of G2 and G3 compared to the control-treated rats within an acceptable normal range on the 45th dosing day and the last dosing day. Interestingly, an increased value of high density lipoprotein and a decreased value of low density lipoprotein were observed in both male and female FLC-treated rats of G2 and G3 compared to the control-treated rats on the last dosing day. The varietions of all biochemical indices of G1, G2 and G3 were not significant on the recovery day compared to control-treated animals.

Hematological observation results are described in Table 3 and Table 4. Small changes were observed in mean corpuscular hemoglobin and red cell distribution width-coefficient of variation in the male FLC-treated rats of G2 and G3 compared to the control-treated rats within an acceptable normal range on the 45th dosing day and the last dosing day.

**Table 1:** Summary of clinical biochemistry in male rats.

Tests	Dosing Days 45				Dosing Days 90				Recovery Days 30			
	G0	G1	G2	G3	G0	G1	G2	G3	G0	G1	G2	G3
	*n* = 6	*n* = 6	*n* = 6	*n* = 6	*n* = 8	*n* = 8	*n* = 8	*n* = 8	*n* = 6	*n* = 6	*n* = 6	*n* = 6
Alanine aminotransferase (U/L)	60 ± 13	54 ± 10	57 ± 16	66 ± 38	70 ± 34	57 ± 10	53 ± 5	45 ± 12	78 ± 11	80 ± 20	76 ± 17	84 ± 40
Alkaline phosphatase (U/L)	99 ± 7	85 ± 8 **	74 ± 4 **	72 ± 5 **	87 ± 15	71 ± 11 *	67 ± 10 **	54 ± 6 **	112 ± 13	106 ± 6	94 ± 12	89 ± 8
Aspartate aminotransferase (U/L)	203 ± 53	177 ± 29	184 ± 38	178 ± 40	178 ± 45	144 ± 29	153 ± 41	120 ± 44 *	209 ± 42	186 ± 58	178 ± 38	189 ± 51
Gamma-glutamyltraspeptidase (U/L)	0.86 ± 0.27	0.72 ± 0.28	0.65 ± 0.24	0.78 ± 0.31	0.59 ± 0.36	0.76 ± 0.20	0.62 ± 0.38	0.73 ± 0.30	0.73 ± 0.20	0.76 ± 0.25	0.73 ± 0.18	0.71 ± 0.18
Total protein (g/L)	58.2 ± 1.7	58.6 ± 1.9	58.4 ± 1.8	58.4 ± 2.8	57.3 ± 2.4	57.3 ± 1.8	59.4 ± 1.2	59.0 ± 1.1	61.1 ± 1.5	59.3 ± 1.2	61.0 ± 1.7	61.0 ± 1.3
Albumin (g/L)	24.4 ± 1.4	25.2 ± 1.2	25.4 ± 1.5	26.2 ± 1.5	24.1 ± 0.9	24.3 ± 0.8	26.0 ± 0.7 **	26.0 ± 1.1 **	24.2 ± 1.5	24.7 ± 0.8	25.3 ± 1.2	25.0 ± 0.7
Creatine phosphokinase (U/L)	1901 ± 617	1659 ± 354	1742 ± 568	1563 ± 341	1632 ± 453	1335 ± 288	1434 ± 680	1105 ± 751	1942 ± 712	1653 ± 807	1384 ± 809	1674 ± 842
Glucose (mmol/L)	6.44 ± 0.97	6.30 ± 1.21	5.66 ± 1.08	5.62 ± 1.26	8.56 ± 3.52	7.49 ± 1.01	7.30 ± 0.86	6.49 ± 1.37	8.46 ± 0.96	8.96 ± 0.99	8.85 ± 1.12	8.66 ± 1.31
Cholesterol (mmol/L)	1.66 ± 0.10	1..75 ± 0.11	1.74 ± 0.19	1.85 ± 0.16	1.80 ± 0.15	1.94 ± 0.19	2.10 ± 0.13 *	1.79 ± 0.14	1.93 ± 0.10	1.87 ± 0.06	2.00 ± 0.21	1.88 ± 0.14
Triglycerides (mmol/L)	0.74 ± 0.24	0.58 ± 0.29	0.43 ± 0.09	0.59 ± 0.13	0.45 ± 0.12	0.55 ± 0.18	0.51 ± 0.13	0.50 ± 0.18	0.86 ± 0.40	0.86 ± 0.21	0.80 ± 0.35	0.84 ± 0.30
Total bilirubin (μmol/L)	0.4 ± 0.2	0.5 ± 0.1	0.4 ± 0.2	0.8 ± 0.2 **	0.5 ± 0.2	0.5 ± 0.2	0.7 ± 0.2	0.7 ± 0.2	0.6 ± 0.3	0.8 ± 0.3	0.8 ± 0.2	0.5 ± 0.3
Urea nitrogen (mmol/L)	6.62 ± 0.62	6.40 ± 0.50	6.62 ± 0.57	6.26 ± 0.49	6.58 ± 0.50	6.32 ± 0.31	6.44 ± 0.49	6.65 ± 0.57	8.28 ± 0.74	7.66 ± 0.65	7.66 ± 0.76	7.44 ± 0.58
Creatinine (μmol/L)	33.6 ± 3.9	31.4 ± 1.6	33.6 ± 1.6	35.5 ± 3.8	36.8 ± 13.0	31.9 ± 2.8	30.8 ± 3.9	32.8 ± 2.4	36.4 ± 4.4	32.6 ± 2.8	36.7 ± 9.1	34.5 ± 8.2
Uric acid (mmol/L)	78.34 ± 10.47	77.47 ± 7.45	80.80 ± 6.97	91.72 ± 28.32	113 ± 109	70.88 ± 19.80	72.57 ± 24.72	61.85 ± 11.77	76.18 ± 21.12	59.56 ± 5.06	91.43 ± 58.05	82.93 ± 55.31
Glycosylated hemoglobin (%) ^#^	/	/	/	/	2.8 ± 0.1	2.8 ± 0.2	2.6 ± 0.1 **	2.1 ± 0.2 **	2.9 ± 0.1	2.4 ± 0.9	2.8 ± 0.1	2.3 ± 1.0
High density lipoprotein (mmol/L) ^#^	/	/	/	/	0.54 ± 0.04	0.55 ± 0.05	0.64 ± 0.05 **	0.57 ± 0.06	0.53 ± 0.05	0.52 ± 0.04	0.54 ± 0.07	0.54 ± 0.05
Low density lipoprotein (mmol/L) ^#^	/	/	/	/	0.08 ± 0.01	0.08 ± 0.01	0.06 ± 0.01	0.06 ± 0.02 *	0.07 ± 0.02	0.06 ± 0.01	0.08 ± 0.02	0.06 ± 0.01
Sodion (mmoL/L)	138.8 ± 1.7	138.9 ± 1.2	139.5 ± 1.4	139.8 ± 1.2	140.3 ± 1.1	140.4 ± 1.2	140.4 ± 1.7	141.0 ± 1.6	138.6 ± 1.5	138.7 ± 1.5	139.1 ± 1.2	139.2 ± 0.6
Potassium ion (mmoL/L)	4.90 ± 0.40	4.98 ± 0.52	4.85 ± 0.39	5.00 ± 0.37	5.09 ± 0.76	5.45 ± 0.88	5.54 ± 0.63	5.25 ± 0.82	6..28 ± 1.10	6.02 ± 0.96	6.95 ± 0.61	6.48 ± 1.01
Chloride ion (mmoL/L)	100.3 ± 0.9	100.3 ± 0.9	100.4 ± 1.5	100.9 ± 1.2	102.1 ± 1.9	102.5 ± 1.9	102.4 ± 1.6	103.1 ± 1.9	99.8 ± 0.9	99.5 ± 0.7	99.4 ± 1.9	99.9 ± 0.9

Notes: G0 means Group 0 (0.5% CMC-Na), G1 means Group 1 (FLC 31.25 mg/kg), G2 means Group 2 (FLC 125 mg/kg), G3 means Group 3 (FLC 500 mg/kg). Data are expressed at mean ± S.D. ^#^ Glycosylated hemoglobin, high density lipoprotein and low density lipoprotein were not detected on the 45th day of dosing phase. * A significant difference at *p* < 0.05 level compared with the control (Group 0). ** A significant difference at *p* < 0.01 level compared with the control (Group 0).

**Table 2:** Summary of clinical biochemistry in female rats.

Tests	Dosing Days 45				Dosing Days 90				Recovery Days 30			
	G0	G1	G2	G3	G0	G1	G2	G3	G0	G1	G2	G3
	*n* = 6	*n* = 6	*n* = 6	*n* = 6	*n* = 8	*n* = 8	*n* = 8	*n* = 8	*n* = 6	*n* = 6	*n* = 6	*n* = 6
Alanine aminotransferase (U/L)	41 ± 4	38 ± 2	37 ± 4	36 ± 5	49 ± 6	46 ± 8	41 ± 10	36 ± 8 *	40 ± 4	42 ± 7	37 ± 5	40 ± 4
Alkaline phosphatase (U/L)	58 ± 11	76 ± 12 *	46 ± 4	42 ± 4 *	56 ± 11	48 ± 11	44 ± 11	30 ± 3 **	53 ± 14	56 ± 2	71 ± 12	59 ± 9
Aspartate aminotransferase (U/L)	134 ± 40	128 ± 28	118 ± 31	109 ± 24	139 ± 33	126 ± 42	129 ± 60	110 ± 25	133 ± 53	141 ± 31	112 ± 33	132 ± 26
Gamma-glutamyltraspeptidase (U/L)	1.04 ± 0.43	1.06 ± 0.07	0.96 ± 0.13	1.05 ± 0.22	0.76 ± 0.31	0.81 ± 0.41	1.01 ± 0.15	0.83 ± 0.36	0.58 ± 0.30	0.72 ± 0.26	0.75 ± 0.22	0.74 ± 0.26
Total protein (g/L)	56.4 ± 2.4	56.6 ± 2.2	58.4 ± 1.9	59.8 ± 1.9	54.4 ± 2.5	56.0 ± 1.8	58.0 ± 1.1 **	59.3 ± 2.5 **	58.4 ± 2.1	58.7 ± 2.6	58.1 ± 1.6	58.8 ± 2.1
Albumin (g/L)	25.5 ± 1.1	26.1 ± 1.0	27.3 ± 0.8 *	28.8 ± 1.2 **	25.7 ± 1.3	26.8 ± 1.3	27.6 ± 1.0 **	29.2 ± 1.0 **	27.1 ± 1.2	27.2 ± 1.1	25.3 ± 0.7	26.4 ± 1.2
Creatine phosphokinase (U/L)	1158 ± 527	1147 ± 411	1003 ± 440	949 ± 391	1268 ± 525	1127 ± 699	1054 ± 582	1158 ± 440	1179 ± 857	1322 ± 487	933 ± 546	1084 ± 589
Glucose (mmol/L)	5.08 ± 0.81	8.19 ± 1.27 **	5.17 ± 0.92	5.44 ± 0.98	7.48 ± 1.25	6.50 ± 1.53	6.98 ± 1.69	5.54 ± 0.39 *	7.37 ± 1.00	7.96 ± 0.90	8.56 ± 1.32	7.26 ± 0.66
Cholesterol (mmol/L)	2.43 ± 0.18	2.32 ± 0.18	2.65 ± 0.23	2.79 ± 0.27	2.37 ± 0.29	2.23 ± 0.20	2.50 ± 0.27	2.99 ± 0.21 **	2.47 ± 0.10	2.19 ± 0.39	2.03 ± 0.22	2.40 ± 0.24
Triglycerides (mmol/L)	0.42 ± 0.06	0.38 ± 0.07	0.39 ± 0.02	0.46 ± 0.08	0.33 ± 0.05	0.35 ± 0.04	0.31 ± 0.08	0.40 ± 0.06	0.39 ± 0.10	0.32 ± 0.05	0.32 ± 0.10	0.32 ± 0.02
Total bilirubin (μmol/L)	0.5 ± 0.2	0.3 ± 0.2	0.6 ± 0.2	0.9 ± 0.2 **	0.3 ± 0.2	0.5 ± 0.3	0.6 ± 0.4	0.8 ± 0.2 *	0.7 ± 0.1	0.6 ± 0.2	0.5 ± 0.3	0.5 ± 0.2
Urea nitrogen (mmol/L)	6.62 ± 0.50	8.26 ± 0.67 **	6.20 ± 0.40	5.96 ± 0.39	7.65 ± 1.46	7.42 ± 0.59	7.35 ± 1.34	6.65 ± 0.41	6.82 ± 0.70	7.46 ± 1.34	8.11 ± 1.01	7.58 ± 0.57
Creatinine (μmol/L)	33.8 ± 3.6	31.8 ± 4.4	31.3 ± 2.1	30.5 ± 3.0	33.8 ± 4.5	33.2 ± 5.9	30.4 ± 3.8	30.0 ± 2.1	34.6 ± 5.4	34.5 ± 3.7	34.4 ± 4.3	32.4 ± 4.1
Uric acid (mmol/L)	78.24 ± 8.60	87.86 ± 22.95	86.12 ± 15.52	81.18 ± 17.07	82.3 ± 33.1	78.9 ± 46.4	60.6 ± 17.7	67.8 ± 13.2	81.4 ± 34.5	76.3 ± 18.6	82.1 ± 32.9	72.9 ± 21.7
Glycosylated hemoglobin (%) ^#^	/	/	/	/	2.4 ± 0.1	2.4 ± 0.1	2.1 ± 0.1 **	1.8 ± 0.1 **	1.8 ± 0.9	2.1 ± 0.9	2.0 ± 0.9	2.1 ± 0.8
High density lipoprotein (mmol/L) ^#^	/	/	/	/	0.78 ± 0.08	0.72 ± 0.08	0.84 ± 0.10	1.02 ± 0.07 **	0.85 ± 0.03	0.76 ± 0.13	0.70 ± 0.07	0.82 ± 0.08
Low density lipoprotein (mmol/L) ^#^	/	/	/	/	0.06 ± 0.02	0.05 ± 0.01	0.05 ± 0.01	0.04 ± 0.01 **	0.03 ± 0.02	0.04 ± 0.02	0.04 ± 0.01	0.05 ± 0.01
High density lipoprotein (mmol/L) ^#^	141.3 ± 1.4	141.6 ± 1.2	141.8 ± 0.6	142.5 ± 2.1	138.7 ± 0.9	122.1 ± 49.4	140.3 ± 1.1	140.0 ± 0.9	139.5 ± 0.8	139.7 ± 1.4	139.7 ± 0.7	139.8 ± 1.4
Low density lipoprotein (mmol/L)^#^	4.86 ± 0.75	5.40 ± 0.72	5.14 ± 0.50	4.78 ± 0.42	5.22 ± 0.63	4.37 ± 1.95	5.22 ± 0.63	5.07 ± 0.60	5.94 ± 0.57	6.15 ± 1.30	6.34 ± 1.22	6.29 ± 0.43
Chloride ion (mmoL/L)	103.2 ± 1.9	104.9 ± 1.9	104.9 ± 0.8	105.4 ± 0.3	101.2 ± 1.3	89.8 ± 36.3	102.0 ± 1.2	101.7 ± 1.0	101.4 ± 0.5	101.2 ± 1.4	102.1 ± 1.3	102.2 ± 0.5

Notes: G0 means Group 0 (0.5% CMC-Na), G1 means Group 1 (FLC 31.25 mg/kg), G2 means Group 2 (FLC 125 mg/kg), G3 means Group 3 (FLC 500 mg/kg). Data are expressed at mean ± S.D. ^#^ Glycosylated hemoglobin, High density lipoprotein and Low density lipoprotein were not detected on the 45th day of dosing phase. * A significant difference at *p* < 0.05 level compared with the control (Group 0). ** A significant difference at *p* < 0.01 level compared with the control (Group 0).

**Table 3:** Summary of hematology in male rats.

Tests	Dosing Days 45				Dosing Days 90				Recovery Days 30			
	G0	G1	G2	G3	G0	G1	G2	G3	G0	G1	G2	G3
	*n* = 6	*n* = 6	*n* = 6	*n* = 6	*n* = 8	*n* = 8	*n* = 8	*n* = 8	*n* = 6	*n* = 6	*n* = 6	*n* = 6
White blood cell (10^9^/L)	8.05 ± 2.12	7.18 ± 2.06	6.45 ± 3.30	8.33 ± 2.38	6.04 ± 1.29	6.81 ± 1.83	5.47 ± 0.90	5.87 ± 1.59	3.43 ± 1.54	2.78 ± 1.11	4.35 ± 1.73	3.07 ± 0.62
Neutrophil (%)	30.9 ± 9.6	26.4 ± 2.5	39.1 ± 17.1	30.0 ± 8.0	31.6 ± 8.2	28.2 ± 5.8	30.2 ± 4.0	27.6 ± 5.7	37.1 ± 6.9	35.8 ± 9.3	30.6 ± 9.6	36.9 ± 7.4
Lymphocytes (%)	63.8 ± 9.6	67.6 ± 2.4	53.2 ± 22.9	63.7 ± 7.1	61.8 ± 8.6	64.8 ± 5.7	63.2 ± 3.3	65.4 ± 5.2	55.3 ± 6.9	55.0 ± 9.9	62.1 ± 8.8	55.2 ± 7.7
Monocyte (%)	2.9 ± 0.4	3.4 ± 1.0	5.6 ± 7.4	3.6 ± 0.7	3.1 ± 0.7	3.3 ± 0.9	2.9 ± 0.5	2.9 ± 1.1	2.4 ± 0.4	3.1 ± 0.6	2.9 ± 0.8	2.7 ± 0.4
Eosinophil (%)	1.4 ± 0.2	1.3 ± 0.2	1.3 ± 0.9	1.4 ± 0.3	1.8 ± 0.3	1.9 ± 0.8	2.0 ± 0.5	2.2 ± 0.6	3.8 ± 2.4	4.0 ± 1.0	2.5 ± 1.8	3.5 ± 0.7
Basophil (%)	0.4 ± 0.1	0.4 ± 0.1	0.3 ± 0.1	0.3 ± 0.1	0.3 ± 0.1	0.3 ± 0.1	0.3 ± 0.1	0.4 ± 0.1	0.5 ± 0.2	0.5 ± 0.2	0.6 ± 0.2	0.4 ± 0.2
Red blood cell (10^12^/ L)	9.41 ± 0.80	9.64 ± 0.20	9.21 ± 0.57	9.48 ± 0.37	9.84 ± 0.35	9.94 ± 0.29	9.98 ± 0.30	9.88 ± 0.28	9.03 ± 0.37	9.03 ± 0.16	9.49 ± 0.19 *	9.38 ± 0.19
Hemoglobin (g/dL)	15.2 ± 1.3	15.8 ± 0.4	14.9 ± 0.8	15.2 ± 0.6	15.7 ± 0.6	16.0 ± 0.6	15.9 ± 0.5	15.5 ± 0.3	15.2 ± 0.5	15.6 ± 0.5	16.0 ± 0.3 *	15.6 ± 0.3
Hematocrit (%)	49.2 ± 3.9	51.1 ± 1.5	48.1 ± 2.5	49.4 ± 2.2	49.0 ± 1.8	49.8 ± 2.3	49.2 ± 1.8	48.8 ± 1.4	45.9 ± 1.8	47.3 ± 1.1	49.0 ± 1.6 *	47.4 ± 1.4
Mean corpuscular volume (fL)	52 ± 0	53 ± 1	52 ± 1	52 ± 1	50 ± 1	50 ± 1	49 ± 1	49 ± 1	51 ± 1	52 ± 1	52 ± 1	51 ± 1
Mean corpuscular hemoglobin (pg)	16.2 ± 0.2	16.4 ± 0.2	16.1 ± 0.4	16.0 ± 0.2	16.0 ± 0.3	16.1 ± 0.1	16.0 ± 0.2	15.7 ± 0.2 *	16.9 ± 0.2	17.1 ± 0.3	16.9 ± 0.1	16.6 ± 0.1
Mean corpuscular hemoglobin concentration (g/dL)	30.9 ± 0.3	31.0 ± 0.3	30.9 ± 0.4	30.7 ± 0.3	32.2 ± 0.4	32.2 ± 0.5	32.4 ± 0.5	31.8 ± 0.4	33.2 ± 0.5	33.0 ± 0.3	32.7 ± 0.7	32.8 ± 0.5
Red cell distribution width-coefficient of variation (%)	11.6 ± 0.2	11.8 ± 0.4	12.2 ± 0.4 *	12.5 ± 0.2 **	11.8 ± 0.3	11.7 ± 0.2	11.7 ± 0.2	12.3 ± 0.4	13.1 ± 0.4	13.2 ± 0.2	13.2 ± 0.3	13.4 ± 0.4
Platelet count (10^9^/L)	1117 ± 286	1171 ± 145	1002 ± 357	1117 ± 95	1241 ± 100	1240 ± 77	1259 ± 84	1226 ± 75	917 ± 197	914 ± 96	1018 ± 60	1027 ± 106
Reticulocytes (%)	2.1 ± 0.2	2.1 ± 0.3	2.2 ± 0.2	2.5 ± 0.3	2.0 ± 0.2	2.1 ± 0.3	2.0 ± 0.3	2.5 ± 0.3 **	2.1 ± 0.5	2.3 ± 0.1	2.2 ± 0.1	2.1 ± 0.2
Reticulocytes count (×10^9^/L)	200.4 ± 15.4	200.4 ± 30.3	206.6 ± 17.1	240.4 ± 24.4 *	196.2 ± 25.4	210.3 ± 31.9	196.6 ± 25.4	247.4 ± 27.9 **	189.9 ± 44.7	213.4 ± 7.9	205.8 ± 12.0	191.3 ± 15.7
Activated partial thromboplastin time (s)	20.4 ± 2.6	19.9 ± 1.5	19.8 ± 2.7	20.3 ± 3.0	29.8 ± 2.4	29.1 ± 3.0	26.6 ± 2.7	26.0 ± 2.3 *	23.1 ± 7.9	20.2 ± 3.5	20.3 ± 1.6	19.9 ± 2.5
Thromboplastin time (s)	50.2 ± 7.4	49.6 ± 6.2	45.7 ± 7.7	40.2 ± 10.8	61.8 ± 7.8	61.2 ± 3.7	59.8 ± 3.0	58.1 ± 3.3	57.2 ± 1.5	54.5 ± 5.3	62.8 ± 3.6	58.5 ± 4.1
Fibrinogen (g/L)	8.7 ± 0.8	8.8 ± 0.8	8.6 ± 0.9	8.5 ± 0.5	10.6 ± 0.9	10.1 ± 0.6	10.2 ± 0.6	10.3 ± 0.9	9.6 ± 2.1	8.7 ± 0.4	8.9 ± 0.9	8.6 ± 0.9
Prothrombin time (s)	8.8 ± 0.4	8.9 ± 0.3	8.8 ± 0.4	8.5 ± 0.3	12.9 ± 0.9	12.4 ± 1.5	11.8 ± 1.2	11.5 ± 1.1	8.9 ± 0.3	8.8 ± 0.3	9.1 ± 0.2	9.0 ± 0.5

Notes: G0 means Group 0 (0.5% CMC-Na), G1 means Group 1 (FLC 31.25 mg/kg), G2 means Group 2 (FLC 125 mg/kg), G3 means Group 3 (FLC 500 mg/kg). Data are expressed at mean ± S.D. * A significant difference at *p* < 0.05 level compared with the control (Group 0). ** A significant difference at *p* < 0.01 level compared with the control (Group 0).

**Table 4:** Summary of hematology in female rats.

Tests	Dosing Days 45				Dosing Days 90				Recovery Days 30			
	G0	G1	G2	G3	G0	G1	G2	G3	G0	G1	G2	G3
	*n* = 6	*n* = 6	*n* = 6	*n* = 6	*n* = 8	*n* = 8	*n* = 8	*n* = 8	*n* = 6	*n* = 6	*n* = 6	*n* = 6
White blood cell (10^9^/L)	4.15 ± 1.15	4.52 ± 0.86	4.88 ± 1.22	4.35 ± 1.60	2.90 ± 0.87	2.57 ± 0.87	3.22 ± 1.11	2.56 ± 1.27	1.95 ± 1.08	2.22 ± 0.97	2.29 ± 0.44	2.09 ± 0.74
Neutrophil (%)	33.0 ± 8.3	30.8 ± 6.8	33.2 ± 5.7	32.6 ± 9.6	29.0 ± 7.4	28.3 ± 8.3	33.3 ± 4.3	25.2 ± 7.0	29.1 ± 3.4	31.8 ± 5.1	35.3 ± 4.2	35.6 ± 12.3
Lymphocytes (%)	61.7 ± 7.8	64.8 ± 6.3	61.6 ± 5.7	62.8 ± 9.6	64.1 ± 5.5	65.3 ± 8.1	60.6 ± 3.9	68.1 ± 6.8	63.3 ± 3.2	60.9 ± 4.2	58.1 ± 4.0	57.2 ± 12.4
Monocyte (%)	3.0 ± 0.7	2.2 ± 0.9	2.6 ± 0.6	2.6 ± 0.8	2.4 ± 1.3	2.7 ± 1.2	2.9 ± 1.3	2.8 ± 0.8	2.0 ± 0.5	2.2 ± 0.8	2.2 ± 0.3	2.2 ± 0.4
Eosinophil (%)	1.3 ± 0.3	1.5 ± 0.4	1.4 ± 0.2	1.7 ± 0.5	3.3 ± 5.0	2.6 ± 1.3	1.5 ± 1.1	1.8 ± 0.5	4.4 ± 1.6	3.8 ± 2.0	3.2 ± 1.2	3.6 ± 0.6
Basophil (%)	0.3 ± 0.1	0.3 ± 0.1	0.3 ± 0.1	0.3 ± 0.2	0.3 ± 0.1	0.3 ± 0.2	0.4 ± 0.1	0.4 ± 0.1	0.6 ± 0.2	0.6 ± 0.3	0.4 ± 0.2	0.5 ± 0.2
Red blood cell (10^12^/ L)	8.84 ± 0.35	8.73 ± 0.27	8.71 ± 0.07	8.40 ± 0.18	8.87 ± 0.22	8.70 ± 0.42	8.79 ± 0.24	8.74 ± 0.37	8.63 ± 0.26	8.79 ± 0.25	8.35 ± 0.29	8.63 ± 0.22
Hemoglobin (g/dL)	15.4 ± 0.7	15.4 ± 0.3	15.1 ± 0.4	14.6 ± 0.4	15.3 ± 0.3	15.0 ± 0.8	14.9 ± 0.4	13.8 ± 3.1	16.0 ± 0.5	16.2 ± 0.6	15.3 ± 0.6	15.7 ± 0.4
Hematocrit (%)	48.6 ± 2.3	47.6 ± 1.4	47.0 ± 0.8	45.4 ± 1.0 **	46.8 ± 1.3	45.8 ± 1.8	46.1 ± 1.3	46.1 ± 1.8	46.8 ± 1.5	47.7 ± 2.0	45.4 ± 1.7	45.9 ± 1.0
Mean corpuscular volume (fL)	55.0 ± 1.0	54.5 ± 0.6	54.0 ± 0.9	54.1 ± 1.1	52.8 ± 0.7	52.7 ± 0.6	52.4 ± 0.8	52.8 ± 0.5	54.2 ± 0.5	54.3 ± 1.0	54.4 ± 1.1	53.2 ± 0.5
Mean corpuscular hemoglobin (pg)	17.4 ± 0.4	17.7 ± 0.3	17.4 ± 0.4	17.4 ± 0.3	17.2 ± 0.1	17.2 ± 0.1	17.0 ± 0.1	15.7 ± 3.2	18.5 ± 0.3	18.4 ± 0.2	18.3 ± 0.2	18.2 ± 0.2
Mean corpuscular hemoglobin concentration (g/dL)	31.6 ± 0.7	32.5 ± 0.5 *	32.1 ± 0.4	32.2 ± 0.2	32.6 ± 0.5	32.6 ± 0.4	32.4 ± 0.5	29.7 ± 6.2	34.2 ± 0.8	34.0 ± 0.4	33.6 ± 1.0	34.2 ± 0.5
Red cell distribution width-coefficient of variation (%)	10.8 ± 0.3	10.6 ± 0.3	11.0 ± 0.6	11.3 ± 0.4	10.1 ± 0.3	10.1 ± 0.2	10.2 ± 0.2	10.4 ± 0.3	11.6 ± 0.4	11.5 ± 0.2	12.0 ± 0.4	11.8 ± 0.2
Platelet count (10^9^/L)	1270 ± 144	1172 ± 199	1088 ± 233	1098 ± 74	1303 ± 75	1134 ± 80 *	1199 ± 72	1090 ± 185 **	1087 ± 137	999 ± 78	1026 ± 68	1100 ± 53
Reticulocytes (%)	2.2 ± 0.3	2.4 ± 0.4	2.5 ± 0.6	2.9 ± 0.7	2.6 ± 0.4	2.7 ± 0.6	2.8 ± 0.3	2.9 ± 0.5	2.2 ± 0.5	1.9 ± 0.2	2.6 ± 1.0	1.9 ± 0.3
Reticulocytes count (×10^9^/L)	193.3 ± 25.6	206.8 ± 31.1	216.0 ± 53.2	243.2 ± 57.9	232.8 ± 35.5	229.7 ± 40.7	245.9 ± 27.9	251.0 ± 43.5	192.4 ± 39.2	170.8 ± 20.0	214.3 ± 82.0	162.7 ± 19.7
Activated partial thromboplastin time (s)	19.0 ± 1.5	20.2 ± 5.1	18.8 ± 3.1	16.9 ± 0.8	24.0 ± 3.0	25.1 ± 2.4	25.3 ± 1.7	24.4 ± 2.1	26.4 ± 2.2	26.1 ± 3.8	24.3 ± 2.9	25.2 ± 1.6
Thromboplastin time (s)	51.6 ± 4.6	44.5 ± 10.3	45.0 ± 6.7	46.3 ± 2.1	52.8 ± 5.0	53.9 ± 3.9	54.2 ± 2.2	52.5 ± 2.6	56.8 ± 3.6	51.0 ± 7.7	55.8 ± 2.8	57.3 ± 1.6
Fibrinogen (g/L)	9.0 ± 0.8	9.9 ± 1.0	9.7 ± 0.9	9.9 ± 0.5	11.4 ± 0.7	11.6 ± 1.0	11.2 ± 1.4	11.5 ± 1.5	12.2 ± 1.7	12.6 ± 0.6	11.1 ± 1.1	11.5 ± 1.3
Prothrombin time (s)	9.0 ± 0.8	8.7 ± 0.4	8.4 ± 0.3	8.4 ± 0.2	10.2 ± 1.1	10.4 ± 1.0	10.7 ± 0.7	10.3 ± 1.0	10.7 ± 1.1	10.3 ± 1.3	10.7 ± 1.6	10.1 ± 0.7

Notes: G0 means Group 0 (0.5% CMC-Na), G1 means Group 1 (FLC 31.25 mg/kg), G2 means Group 2 (FLC 125 mg/kg), G3 means Group 3 (FLC 500 mg/kg). Data are expressed at mean ± S.D. * A significant difference at *p* < 0.05 level compared with the control (Group 0). ** A significant difference at *p* < 0.01 level compared with the control (Group 0).

In addition, small changes were observed in hematocrit and mean corpuscular hemoglobin concentration in the female FLC-treated rats of G1 and G3 compared to the control-treated rats within an acceptable normal range on the 45th dosing day. A significantly decreased platelet count value was observed in the female FLC-treated rats of G1 and G3 compared to the control-treated rats on the last dosing day. A significantly increased value of reticulocytes was observed in the male FLC-treated rats of G3 compared to the control-treated rats on the 45th dosing day and the last dosing day. Significantly increased values of red blood cell, hemoglobin and hematocrit counts were observed in the male FLC-treated rats of G2 compared to the control-treated rats on the last recovery day. The variation of all the hematology indices of female rats in G1, G2 and G3 were not significant on the last recovery day compared to control-treated animals. A decreased value of activated partial thromboplastin time was observed in the male FLC-treated rats of G3 compared to the control-treated rats on the last dosing day. In addition, no significant changes of thromboplastin time, fibrinogen and prothrombin time were observed in the coagulation parameters in any group compared with the control-treated rats (Table 3 and Table 4). The variation of all coagulation indices of G1, G2 and G3 were not significant on the last recovery day compared to control-treated animals. There were no alterations in the routine urine tests on the 45th dosing day, the last dosing day and the last day of the recovery phase.

### 3.3. Endocrine Function

A significantly increased value of T in serum was observed in the male FLC-treated rats of Group 3 compared to the control-treated rats on the last dosing day. A similar trend was also shown on the 45th day. The T level differences of G1, G2 and G3 were not significant on the last recovery day compared to control-treated animals. In addition, no treatment-related alterations were observed in the levels of E2, FSH, LH on the 45th day and the last dosing day (Table 5 and Table 6).

### 3.4. Necropsy and Histopathlolgoy

Organs coefficients are given in Table 7 and Table 8. A significantly increased value of brain coefficients was observed in the male FLC-treated rats of G2 and G3 compared with the control-treated rats on the last dosing day. A significantly increased value of heart coefficients was observed in the male FLC-treated rats of G3 on the 45th dosing day. The same trend of heart coefficients was observed in the female FLC-treated rats of G2 and G3 on the last dosing day. A significantly increased value of liver coefficient was observed in male and female FLC-treated rats of almost all groups on the 45th dosing day and the last dosing day. Female rats of G3 exhibited increases in kidney coefficient value on the last dosing day. A similar trend of thymus coefficients was observed in the female FLC-treated rats of G3 on the last recovering day. In addition, male rats who received FLC exhibited a decreased dependence on dose concerning the value of testis and epididymis coefficients in all groups compared with the control treated rats on the 45th dosing day, the last dosing day and the last recovery day. By the 45th day, FLC administration produced prolonged or severe germ cell degeneration in all male rats treated with FLC, as well as severe absence of most or all germ cells from affected tubules, severe tubules lined only by sertoli cells, severe decreased tubular diameter, decreased testis size and weight in G2 and G3 male rats.

**Table 5:** Summary of hormone in serum in male rats.

Tests	Dosing Day 45				Dosing Day 90				Recovery Day 30			
	G0	G1	G2	G3	G0	G1	G2	G3	G0	G1	G2	G3
	*n* = 6	*n* = 6	*n* = 6	*n* = 6	*n* = 8	*n* = 8	*n* = 8	*n* = 8	*n* = 6	*n* = 6	*n* = 6	*n* = 6
Testosterone (T, ng/mL)	1.64 ± 1.34	2.25 ± 1.56	2.26 ± 1.68	3.02 ± 1.20	0.71 ± 0.40	1.07 ± 0.40	0.74 ± 0.15	1.31 ± 0.44 *	0.95 ± 0.24	0.92 ± 0.14	0.94 ± 0.34	0.94 ± 0.17
Follicle-Stimulating Hormone (FSH, mIU/mL)	1.89 ± 0.44	2.10 ± 0.48	2.48 ± 0.45	2.20 ± 0.68	2.46 ± 0.81	3.05 ± 0.65	2.79 ± 0.79	2.63 ± 0.59	2.53 ± 0.84	2.45 ± 0.85	2.28 ± 0.88	2.15 ± 1.06
Luteinizing Hormone (LH, mIU/mL)	5.02 ± 1.19	3.75 ± 1.33	4.31 ± 0.59	4.24 ± 1.46	3.68 ± 1.05	4.05 ± 0.60	3.68 ± 1.06	3.24 ± 0.72	6.51 ± 0.85	5.84 ± 2.77	7.70 ± 1.18	5.56 ± 2.28

Notes: G0 means Group 0 (0.5% CMC-Na), G1 means Group 1 (FLC 31.25 mg/kg), G2 means Group 2 (FLC 125 mg/kg), G3 means Group 3 (FLC 500 mg/kg). Data are expressed at mean ± S.D. * A significant difference at *p* < 0.05 level compared with the control (Group 0).

**Table 6:** Summary of hormone in serum in female rats.

Tests	Dosing Day 45				Dosing Day 90				Recovery Day 30			
	G0	G1	G2	G3	G0	G1	G2	G3	G0	G1	G2	G3
	*n* = 6	*n* = 6	*n* = 6	*n* = 6	*n* = 8	*n* = 8	*n* = 8	*n* = 8	*n* = 6	*n* = 6	*n* = 6	*n* = 6
Estradiol (E2, pg/mL)	23.32 ± 11.34	17.20 ± 7.06	24.66 ± 8.82	27.14 ± 16.43	21.88 ± 13.20	20.32 ± 22.18	21.06 ± 14.68	21.84 ± 14.99	24.64 ± 12.99	25.38 ± 12.49	26.74 ± 11.42	26.46 ± 14.27
Follicle-Stimulating Hormone (FSH, mIU/mL)	3.07 ± 0.83	2.83 ± 0.51	3.07 ± 0.70	3.07 ± 0.74	2.50 ± 1.00	2.29 ± 0.82	3.00 ± 0.49	2.75 ± 0.93	2.08 ± 0.66	2.04 ± 0.20	2.17 ± 0.89	1.84 ± 0.42
Luteinizing Hormone (LH, mIU/mL)	6.61 ± 0.95	6.08 ± 0.92	6.22 ± 1.09	5.80 ± 0.98	4.52 ± 0.93	4.21 ± 1.21	4.60 ± 0.66	4.64 ± 0.82	6.84 ± 1.23	4.99 ± 2.10	6.50 ± 2.47	6.90 ± 1.07

Notes: G0 means Group 0 (0.5% CMC-Na), G1 means Group 1 (FLC 31.25 mg/kg), G2 means Group 2 (FLC 125 mg/kg), G3 means Group 3 (FLC 500 mg/kg). Data are expressed at mean ± S.D.

**Table 7:** Summary of organ coefficients in male rats.

Tests	Dosing Day 45				Dosing Day 90				Recovery Day 30			
	G0	G1	G2	G3	G0	G1	G2	G3	G0	G1	G2	G3
	*n* = 6	*n* = 6	*n* = 6	*n* = 6	*n* = 8	*n* = 8	*n* = 8	*n* = 8	*n* = 6	*n* = 6	*n* = 6	*n* = 6
Brain ratio	0.5176 ± 0.0433	0.5278 ± 0.0163	0.5261 ± 0.0295	0.5471 ± 0.0320	0.4665 ± 0.0212	0.5009 ± 0.0340	0.5154 ± 0.0272 **	0.5038 ± 0.0180 *	0.4631 ± 0.05178	0.4527 ± 0.03946	0.4493 ± 0.0387	0.4675 ± 0.0193
Heart ratio	0.3070 ± 0.0133	0.3255 ± 0.0152	0.3221 ± 0.0233	0.3616 ± 0.0526 *	0.3021 ± 0.0224	0.3099 ± 0.0236	0.3070 ± 0.0220	0.3114 ± 0.0132	0.3038 ± 0.0354	0.3002 ± 0.0290	0.2949 ± 0.0269	0.3184 ± 0.0108
Liver ratio	2.5030 ± 0.0957	2.7033 ± 0.1136 **	2.8729 ± 0.0761 **	3.2967 ± 0.0817 **	2.2965 ± 0.1131	2.5475 ± 0.0717 **	2.6937 ± 0.1213 **	2.9500 ± 0.1618 **	2.3125 ± 0.4473	2.5547 ± 0.2937	2.4687 ± 0.1151	2.5664 ± 0.0952
Lung ratio	0.3660 ± 0.0101	0.3730 ± 0.0206	0.4137 ± 0.0391	0.3865 ± 0.0274	0.3416 ± 0.0216	0.3500 ± 0.0279	0.3613 ± 0.0214	0.3832 ± 0.0455	0.3515 ± 0.0397	0.3447 ± 0.0273	0.3264 ± 0.0393	0.3371 ± 0.0291
Kidney ratio	0.6124 ± 0.0820	0.5709 ± 0.0510	0.5834 ± 0.0142	0.6121 ± 0.0173	0.5491 ± 0.0667	0.5957 ± 0.0739	0.5798 ± 0.0258	0.5714 ± 0.0232	0.5574 ± 0.0688	0.5334 ± 0.0547	0.5250 ± 0.1694	0.5617 ± 0.0189
Adrenal gland ratio	0.0136 ± 0.0014	0.0128 ± 0.0027	0.0144 ± 0.0017	0.0148 ± 0.0028	0.0125 ± 0.0017	0.0113 ± 0.0028	0.0109 ± 0.0014	0.0107 ± 0.0016	0.0121 ± 0.0025	0.0108 ± 0.0011	0.0100 ± 0.0032	0.0141 ± 0.0030
Thymus ratio	0.1182 ± 0.0111	0.1163 ± 0.0176	0.1145 ± 0.0164	0.1066 ± 0.0071	0.0834 ± 0.0094	0.0915 ± 0.0155	0.0828 ± 0.0148	0.0810 ± 0.0242	0.0695 ± 0.0129	0.0794 ± 0.0140	0.0736 ± 0.0098	0.0824 ± 0.0127
Spleen ratio	0.1740 ± 0.0083	0.1731 ± 0.0073	0.1753 ± 0.0158	0.1762 ± 0.0165	0.1628 ± 0.0129	0.1725 ± 0.0118	0.1655 ± 0.0075	0.1729 ± 0.0133	0.1611 ± 0.0167	0.1594 ± 0.0151	0.1606 ± 0.0140	0.1628 ± 0.0095
Testis ratio	0.9201 ± 0.0543	0.3667 ± 0.0944 **	0.2832 ± 0.1246 **	0.3547 ± 0.0497 **	0.8318 ± 0.0441	0.2618 ± 0.1022 **	0.2792 ± 0.0762 **	0.3764 ± 0.0982 **	0.7346 ± 0.1516	0.2468 ± 0.0862 **	0.2541 ± 0.0529 **	0.2679 ± 0.0250 **
Epididymis ratio	0.2684 ± 0.0303	0.1822 ± 0.0362 **	0.1928 ± 0.0321 **	0.1666 ± 0.0310 **	0.2822 ± 0.0346	0.1901 ± 0.0311 **	0.1527 ± 0.0220 **	0.1755 ± 0.0497 **	0.2485 ± 0.0269	0.1687 ± 0.01770 **	0.1480 ± 0.0151 **	0.1669 ± 0.0307 **
Prostate ratio	0.1063 ± 0.0360	0.1217 ± 0.0174	0.0960 ± 0.0115	0.0972 ± 0.0183	0.1945 ± 0.0426	0.1863 ± 0.0445	0.2212 ± 0.0662	0.1766 ± 0.0478	0.1186 ± 0.0325	0.1305 ± 0.0312	0.0985 ± 0.0329	0.0875 ± 0.0210
Seminal vesicle ratio	0.2229 ± 0.0773	0.2623 ± 0.0734	0.2301 ± 0.0788	0.1868 ± 0.0599	0.2183 ± 0.0743	0.1998 ± 0.0516	0.2121 ± 0.0644	0.2643 ± 0.1102	0.2191 ± 0.0613	0.2183 ± 0.0425	0.2474 ± 0.0949	0.2167 ± 0.0878

Notes: G0 means Group 0 (0.5% CMC-Na), G1 means Group 1 (FLC 31.25 mg/kg), G2 means Group 2 (FLC 125 mg/kg), G3 means Group 3 (FLC 500 mg/kg). Data are expressed at mean ± S.D. * A significant difference at *p* < 0.05 level compared with the control (Group 0). ** A significant difference at *p* < 0.01 level compared with the control (Group 0).

**Table 8:** Summary of organ coefficients in female rats.

Tests	Dosing Day 45				Dosing Day 90				Recovery Day 30			
	G0	G1	G2	G3	G0	G1	G2	G3	G0	G1	G2	G3
	*n* = 6	*n* = 6	*n* = 6	*n* = 6	*n* = 8	*n* = 8	*n* = 8	*n* = 8	*n* = 6	*n* = 6	*n* = 6	*n* = 6
Brain ratio	0.8160 ± 0.0327	0.8111 ± 0.0434	0.8240 ± 0.0545	0.8565 ± 0.0443	0.7515 ± 0.0478	0.7575 ± 0.0407	0.8112 ± 0.0734	0.8168 ± 0.0645	0.7414 ± 0.0224	0.7473 ± 0.0461	0.7338 ± 0.0683	0.7930 ± 0.0486
Heart ratio	0.3498 ± 0.0246	0.3532 ± 0.0312	0.3597 ± 0.0266	0.3563 ± 0.0183	0.3275 ± 0.0177	0.3383 ± 0.0098	0.3745 ± 0.0394 *	0.3736 ± 0.0206 **	0.3347 ± 0.0244	0.3403 ± 0.0081	0.3466 ± 0.0065	0.3526 ± 0.0080
Liver ratio	2.4911 ± 0.1233	2.6718 ± 0.2241	2.7763 ± 0.1911 *	3.2757 ± 0.0632 **	2.3098 ± 0.1340	2.4276 ± 0.0913	2.8209 ± 0.3459 *	3.1955 ± 0.1947 **	2.4413 ± 0.1328	2.4674 ± 0.3438	2.6624 ± 0.4890	2.6088 ± 0.2043
Lung ratio	0.4677 ± 0.0496	0.4340 ± 0.0160	0.4747 ± 0.0614	0.4747 ± 0.0324	0.4081 ± 0.0212	0.4116 ± 0.0261	0.4225 ± 0.1007	0.4550 ± 0.0188	0.4258 ± 0.0201	0.3946 ± 0.0197	0.4273 ± 0.0329	0.4593 ± 0.0299
Kidney ratio	0.6407 ± 0.0346	0.6640 ± 0.0283	0.6562 ± 0.0261	0.6625 ± 0.0216	0.6060 ± 0.0226	0.6131 ± 0.0254	0.6978 ± 0.0898	0.6982 ± 0.0148 **	0.6209 ± 0.0109	0.6131 ± 0.0466	0.6454 ± 0.0512	0.6535 ± 0.0296
Adrenal gland ratio	0.0253 ± 0.0037	0.0255 ± 0.0034	0.0247 ± 0.0036	0.0224 ± 0.0098	0.0209 ± 0.0019	0.0204 ± 0.0030	0.0234 ± 0.0036	0.0225 ± 0.0017	0.0228 ± 0.0033	0.0186 ± 0.0051	0.0230 ± 0.0032	0.0256 ± 0.0030
Thymus ratio	0.1509 ± 0.0225	0.1581 ± 0.0202	0.1548 ± 0.0221	0.1346 ± 0.0122	0.1211 ± 0.0097	0.1204 ± 0.0189	0.1356 ± 0.0415	0.1089 ± 0.0186	0.1092 ± 0.0097	0.1194 ± 0.0103	0.1137 ± 0.0206	0.1436 ± 0.0038 **
Spleen ratio	0.2153 ± 0.0246	0.1951 ± 0.0112	0.2090 ± 0.0237	0.1924 ± 0.0151	0.1853 ± 0.0080	0.1933 ± 0.0167	0.2036 ± 0.0222	0.1902 ± 0.0081	0.1912 ± 0.0138	0.1833 ± 0.0167	0.2061 ± 0.0173	0.2101 ± 0.0125
Ovary ratio	0.2207 ± 0.0428	0.2290 ± 0.0409	0.2239 ± 0.0359	0.2313 ± 0.0256	0.0188 ± 0.0021	0.0191 ± 0.0028	0.0227 ± 0.0047	0.0208 ± 0.0035	0.0194 ± 0.0046	0.0211 ± 0.0051	0.0213 ± 0.0034	0.0249 ± 0.0026
Uterus ratio	0.1613 ± 0.0444	0.1665 ± 0.0380	0.1775 ± 0.0362	0.1802 ± 0.0396	0.1661 ± 0.0377	0.1647 ± 0.0450	0.1821 ± 0.0419	0.1763 ± 0.0428	0.1957 ± 0.0202	0.1512 ± 0.0315	0.1971 ± 0.0515	0.1570 ± 0.0287
Fallopian tube ratio	0.8107 ± 0.2994	0.8223 ± 0.1068	0.8534 ± 0.2957	1.0718 ± 0.1676	0.0115 ± 0.0040	0.0092 ± 0.0008	0.0115 ± 0.0035	0.0102 ± 0.0023	0.0094 ± 0.0018	0.0105 ± 0.0023	0.0111 ± 0.0024	0.0123 ± 0.0021

Notes: G0 means Group 0 (0.5% CMC-Na), G1 means Group 1 (FLC 31.25 mg/kg), G2 means Group 2 (FLC 125 mg/kg), G3 means Group 3 (FLC 500 mg/kg). Data are expressed at mean ± S.D. * A significant difference at *p* < 0.05 level compared with the control (Group 0). ** A significant difference at *p* < 0.01 level compared with the control (Group 0).

**Figure 3:**
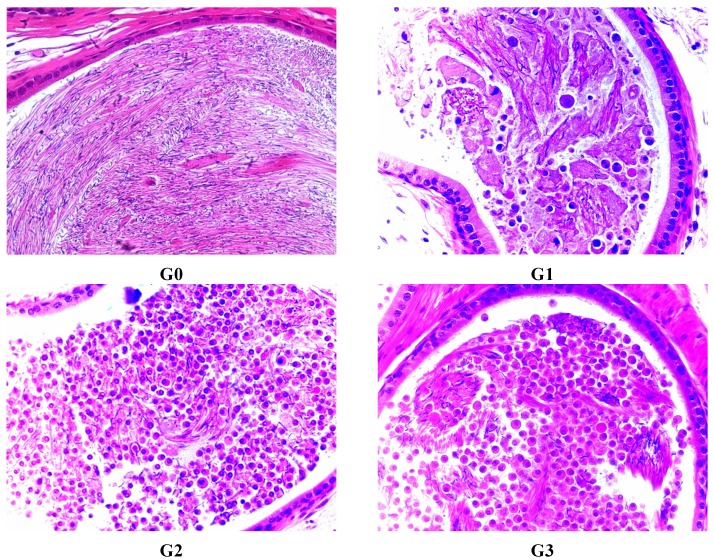
Epididymis lesions in male rats treated with FLC on day 45 for exfoliation and sloughing of testicular germ cells into the epididymal lumen, stained with H&E. G0 means Group 0 (0.5% CMC-Na) , G1 means Group 1 (FLC 31.25 mg/kg), G2 means Group 2 (FLC 125 mg/kg), G3 means Group 3 (FLC 500 mg/kg).

G1 male rats presented a small number of residual germ and sperm cells in affected tubules. There were very few sloughed germ cells in the epididymis of all male rats treated with FLC. Cell debris were present in the epididymal lumen. There were obvious testis and epididymis lesion differences among G1, G2 and G3, indicating a dose dependent toxicity in rats (Figure 2 and Figure 3, Table 9).

**Table 9:** Summary of histopathological findings in male rats.

	Dosing Day 45				Dosing Day 90				Recovery Day 30			
	G0	G1	G2	G3	G0	G1	G2	G3	G0	G1	G2	G3
	*n* = 6	*n* = 6	*n* = 6	*n* = 6	*n* = 8	*n* = 8	*n* = 8	*n* = 8	*n* = 6	*n* = 6	*n* = 6	*n* = 6
**Testis**												
***Atrophy***	0	6	6	6	0	8	8	8	0	6	6	6
Unremarkable (−)	6	0	0	0	8	0	0	0	6	0	0	0
Minimal (+1)	0	0	0	0	0	0	0	0	0	0	0	0
Mild (+2）	0	0	0	0	0	0	0	0	0	0	0	0
Moderate (+3)	0	3	0	0	0	3	0	0	0	6	0	0
Marked (+4)	0	3	0	0	0	5	0	0	0	0	0	0
Severe (+5)	0	0	6	6	0	0	8	8	0	0	6	6
***Degeneration***	0	6	6	6	0	8	8	8	0	6	6	6
Unremarkable (−)	6	0	0	0	8	0	0	0	6	0	0	0
Minimal(+1)	0	0	0	0	0	0	0	0	0	0	0	0
Mild (+2)	0	1	0	0	0	0	0	0	0	0	0	0
Moderate (+3)	0	4	0	0	0	3	0	0	0	6	0	0
Marked (+4)	0	1	1	0	0	5	0	0	0	0	0	0
Severe (+5)	0	0	5	6	0	0	8	8	0	0	6	6
***Fibrosis***	0	2	5	4	0	5	6	6	0	6	6	6
Unremarkable (−)	6	4	1	2	8	3	2	2	6	0	0	0
Minimal (+1)	0	2	1	0	0	5	3	2	0	2	0	0
Mild (+2)	0	0	4	4	0	0	3	4	0	3	0	0
Moderate (+3)	0	0	0	0	0	0	0	0	0	1	2	0
Marked (+4)	0	0	0	0	0	0	0	0	0	0	4	6
Severe (+5)	0	0	0	0	0	0	0	0	0	0	0	0
**Epididymis**												
***germ cell debris***	0	6	6	6	0	8	8	8	0	6	6	6
Unremarkable (−)	6	0	0	0	8	0	0	0	6	0	0	0
Minimal (+1)	0	0	0	0	0	0	0	0	0	0	0	5
Mild (+2)	0	0	0	0	0	1	0	0	0	3	2	0
Moderate (+3)	0	6	3	2	0	6	1	0	0	3	4	1
Marked (+4)	0	0	3	4	0	1	6	6	0	0	0	0
Severe (+5)	0	0	0	0	0	0	1	2	0	0	0	0
***Reduced sperm***	0	6	6	6	0	8	8	8	0	6	6	6
Unremarkable (−)	6	0	0	0	8	0	0	0	6	0	0	0
Minimal (+1)	0	0	0	0	0	0	0	0	0	0	0	0
Mild (+2)	0	0	0	0	0	1	0	0	0	1	0	0
Moderate (+3)	0	5	0	0	0	5	0	0	0	5	0	0
Marked (+4)	0	1	0	0	0	2	1	0	0	0	2	0
Severe (+5)	0	0	6	6	0	0	7	8	0	0	4	6

Notes: G0 means Group 0 (0.5% CMC-Na), G1 means Group 1 (FLC 31.25 mg/kg), G2 means Group 2 (FLC 125 mg/kg), G3 means Group 3 (FLC 500 mg/kg).

**Figure 4:**
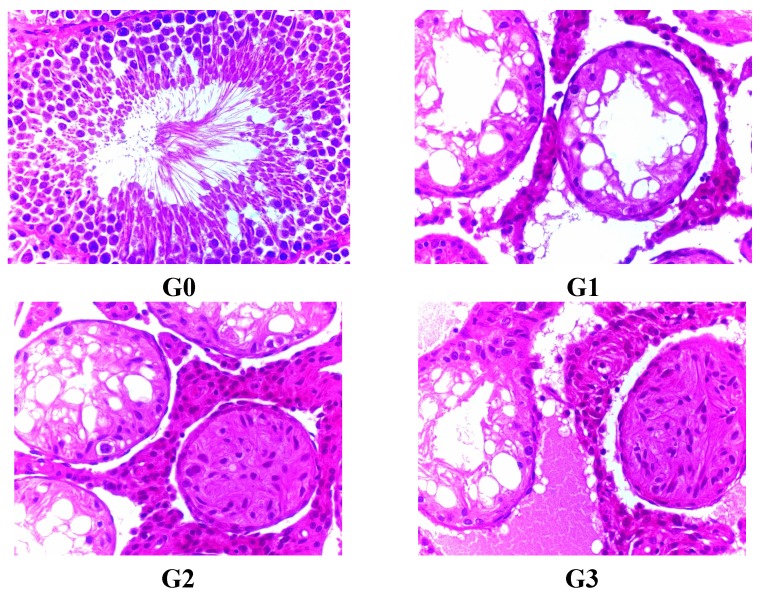
Testis lesions in male rats treated with FLC after recovery for atrophic and degenerating tubules. There were a little of fibrotic tubules in G2 and G3, stained with H&E. G0 means Group 0 (0.5% CMC-Na), G1 means Group 1 (FLC 31.25 mg/kg), G2 means Group 2 (FLC 125 mg/kg), G3 means Group 3 (FLC 500 mg/kg).

**Figure 5:**
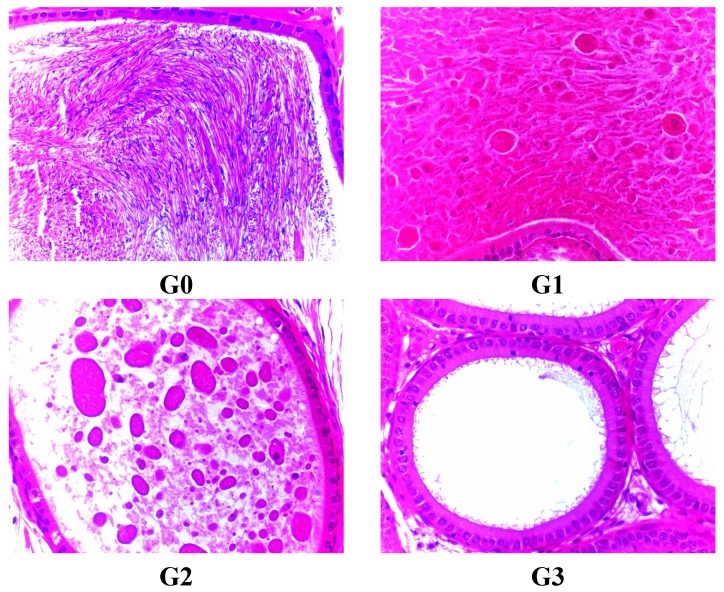
Epididymis lesions in male rats treated with FLC after recovery for exfoliation and sloughing of testicular germ cell debris into the epididymal lumen. There were nothing in the epididymal lumen in G3, stained with H&E. G0 means Group 0 (0.5% CMC-Na), G1 means Group 1 (FLC 31.25 mg/kg), G2 means Group 2 (FLC 125 mg/kg), G3 means Group 3 (FLC 500 mg/kg).

No lesions affecting Leydig cells were observed in any groups treated with FLC. Main organs did not present any lesion in any of the groups. On the last dosing day, there were no significant differences in testis and epididymis lesions compared to the 45th day. After the recovery phase, the degree of toxicity of FLC on testis and epididymis was not relieved (Figure 4 and Figure 5).

### 3.5. Toxicokinetic Evaluation in Rats

**Figure 6:**
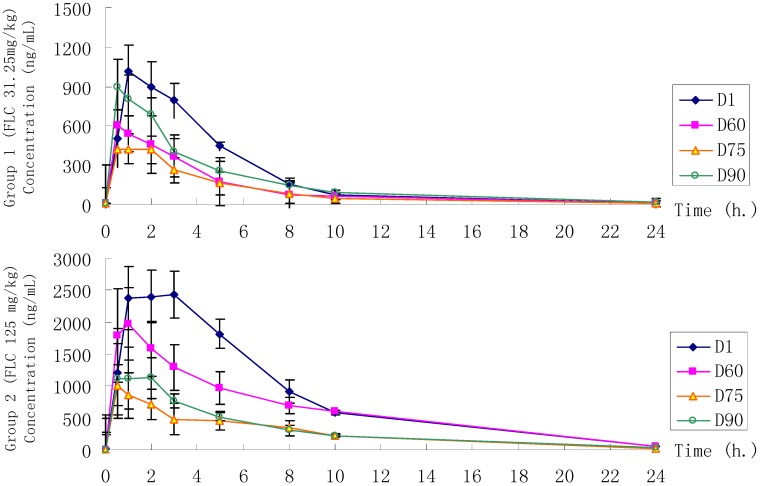

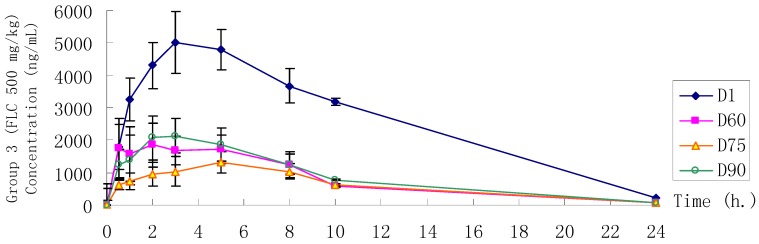
Mean plasma concentration-time profiles of FLC by oral administration in Group 1, Group 2 and Group 3. Group 1 (FLC 31.25 mg/kg), Group 2 (FLC 125 mg/kg) and Group 3 (FLC 500 mg/kg).

**Figure 7:**
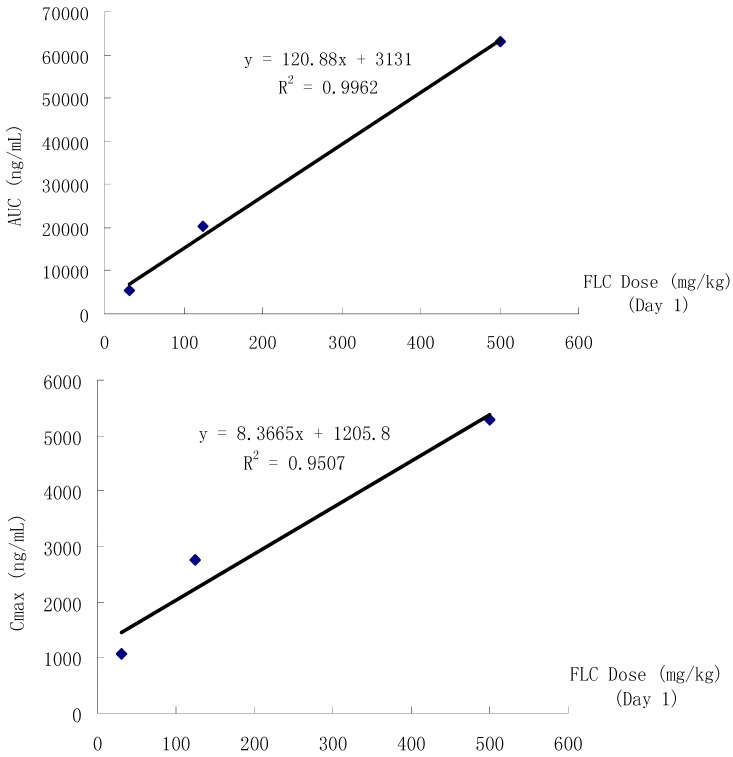
The linear relationship between FLC dose and AUC or C_max_ on the first day.

Plasma concentration-time profiles and linear relation of FLC in rats are shown in Figure 6 and Figure 7. Corresponding exposure data in terms of C_max_, T_max_ and AUC of FLC in male and female rats are summarized in Table 10 and Table 11, respectively. As shown in Figure 6 and Figure 7, FLC could be detected in all animal groups during the dosing phase, and there was a dose proportional relationship between FLC dose and AUC or C_max_ on the 1st dosing day. There were significant differences of C_max_ and AUC among the three groups which indicated a dose dependent relationship on the 1st, 60th, 75th, and 90th day. The toxicokinetic profiles of FLC exhibited analogous variation trends in all groups. AUC and C_max_ both decreased on the 60th, 75th, and 90th day of the dosing phase. In G1, G2 and G3, on the 75th day compared to the 1st day, repeated doses of FLC produced approximately 55%, 68%, 76% lower AUC, 54%, 60%, 73% lower C_max_, and 122%, 215%, 350% higher CLz/F, respectively, but on the last dosing day compared to the 1st day, repeated doses of FLC produced approximately 20%, 61%, 66% lower AUC, 10%, 56%, 58% lower C_max_, and 28%, 181%, 218% higher CLz/F, respectively. The decreasing toxicokinetic manifestation of AUC and C_max_ might be associated with the increasing CLz/F. After oral administration, FLC was absorbed, and the average T_max_ for low-, middle- and high-doses were 1.8 h, 2.4 h and 4.1 h on the 1st day. The average T_max_ in G1 and G2 were decreasing on the 60th, 75th, and 90th day compared to the 1st day, but the average T_max_ in G3 was increasing compared to the 1st day at the same time.

**Table 10:** Toxicokinetic parameters of FLC in male rats.

Tests	Day 1			Day 60			Day 75			Day 90		
	G1	G2	G3	G1	G2	G3	G1	G2	G3	G1	G2	G3
AUC_(0–24 h)_ (ng·h/mL)	5915 ± 376	21,528 ± 2435	62,261 ± 3458	2817 ± 576	15,608 ± 2086	15,629 ± 4108	2810 ± 255	7355 ± 1555	14,098 ± 2666	4315 ± 953	6973 ± 1136	18,766 ± 3281
C_max_ (ng/mL)	1064.0 ± 117.7	2574.0 ± 423.6	5708.0 ± 503.9	557.9 ± 149.3	1640.0 ± 191.0	1942.0 ± 593.4	436.6 ± 133.0	1019.6 ± 271.4	1634.0 ± 380.4	767.2 ± 97.0	830.4 ± 243.4	1988.0 ± 509.0
T_max_ (h)	1.8 ± 1.1	3.0 ± 0.0	3.8 ± 1.1	0.7 ± 0.3	2.1 ± 1.7	5.8 ± 2.2	1.5 ± 1.0	1.2 ± 0.8	5.0 ± 0.0	1.0 ± 0.6	1.7 ± 0.7	3.8 ± 2.4
CLz/F (L/h/kg)	5.3 ± 0.3	5.8 ± 0.7	7.7 ± 1.1	10.8 ± 2.6	7.9 ± 1.2	32.5 ± 7.9	11.1 ± 0.9	17.3 ± 3.7	35.7 ± 5.5	7.2 ± 1.6	17.3 ± 2.7	26.8 ± 5.2

Notes: G1 means Group 1 (FLC 31.25 mg/kg), G2 means Group 2 (FLC 125 mg/kg), G3 means Group 3 (FLC 500 mg/kg). Data are expressed at mean ± S.D. (*n* = 5).

**Table 11:** Toxicokinetic parameters of FLC in female rats.

Tests	Day 1			Day 60			Day 75			Day 90		
	G1	G2	G3	G1	G2	G3	G1	G2	G3	G1	G2	G3
AUC_(0–24 h)_ (ng·h/mL)	4674 ± 324	18,989 ± 2039	64,072 ± 3808	2817 ± 1013	14,910 ± 4053	21,372 ± 6080	1971 ± 177	5316 ± 542	15,188 ± 1912	4108 ± 1198	8874 ± 1061	24,265 ± 5413
C_max_ (ng/mL)	1046.8 ± 262.2	2960.0 ± 512.0	4865.0 ± 435.6	756.0 ± 140.4	2452.0 ± 745.5	2558.0 ± 781.3	522.0 ± 62.2	1210.2 ± 242.6	1210.0 ± 151.8	1122.6 ± 275.1	1658.5 ± 351.5	2818.0 ± 711.3
T_max_ (h)	1.0 ± 0.0	1.8 ± 1.1	4.2 ± 2.5	0.7 ± 0.3	1.0 ± 0.0	6.6 ± 3.2	1.1 ± 0.8	0.5 ± 0.0	5.2 ± 2.8	0.6 ± 0.2	1.4 ± 0.8	3.6 ± 2.5
CLz/F (L/h/kg)	6.7 ± 0.5	5.3 ± 0.7	7.4 ± 0.5	12.1 ± 4.0	7.1 ± 1.8	22.7 ± 4.1	15.8 ± 1.3	22.9 ± 1.7	32.4 ± 4.3	8.1 ± 2.1	14.0 ± 2.1	21.3 ± 6.4

Notes: G1 means Group 1 (FLC 31.25 mg/kg), G2 means Group 2 (FLC 125 mg/kg), G3 means Group 3 (FLC 500 mg/kg). Data are expressed at mean ± S.D. (*n* = 5).

## 4. Discussion

EFSA has reported that oral administration of FLC could affect the testis, epididymis, heart, great vessels and haematopoietic system in rats [7]. However, there is little available technical data about the systemic toxicity and toxicokinetics of FLC after repeated administration. In this work we aimed to provide a sound basis for the safety evaluation of FLC.

The results of our studies demonstrated that 90 days of repeated administration of FLC could cause severe toxicity to the testis and epididymis, obvious toxicity to the liver and hematopoietic system, and potential toxicity to the brain, heart, pancreas, kidney and thymus. There were no obvious signs to suggest toxicity to the great vessels. Testis and epididymis lesion from the microscopic examination combined with decreased organ coefficients showed a severe toxicity of FLC to the reproductive organs in male rats. What is interesting is that the level of testosterone was increasing in male rats treated with high doses of FLC, but different sperm cell stages were not observed in the seminiferous tubules. Testosterone is secreted by Leydig cells and the adrenal gland to assist in completing spermatogenesis. Leydig cell lesions were not apparent from the microscopic examination. It was reported that testosterone could decrease the level of HDL-C [15,16,17,18], but increased levels of HDL-C were observed in both male and female rats. Testosterone also could improve insulin sensitivity [15,19,20]. This finding suggested that FLC could be an endocrine disrupter. Further studies should be performed to clarify the toxicity and mechanism of action of FLC on the testis, epididymis, pancreas, vessels, *etc.* Tubular atrophy is an end-stage lesion where there are no germ cells left within a tubule. It can result from progressive degeneration and phagocytosis/exfoliation of germ cells or cumulative depletion of germ cells. It is a treatment-related change. A greater number are present in the epididymis in all male rats treated with FLC, due to the inefficiency of the first cycle of spermatogenesis. Increased numbers of sloughed, degenerate germ cells in the epididymal lumen of male rats treated with FLC generally reflect ongoing severe testicular degeneration/atrophy, spermatid degeneration, or germ cell exfoliation. The location of the cell debris can provide important information on when cells were sloughed from the testes. Cell debris in the caput would have been released from the testis within the previous 3 days whereas cells in the distal cauda were probably released more than a week previously. The presence of cell debris in the epididymis is often associated with increased prominence of clear cells in the distal corpus and cauda, probably due to increased endocytosis of particulate matter. The decreased testis and epididymis coefficients in all FLC-treated groups without a recovery trend revealed that the oral NOAEL of the 90 day toxicity study could be much less than 31.25 mg/kg according to the histopathological findings. This finding is similar to the EFSA report. Previous work on FLC has suggested that the compound interferes with mitosis. The current findings suggested toxicity to organs or cell grouping which were very mitotically active like the seminiferous tubules (spermatogonia to primary spermatocytes) and liver.

The toxicokinetic findings provided a clue to understand the influence of the pattern of repeated exposure to FLC in rats on the absorption phase. FLC (31.25–500 mg/kg) showed a persistent plasma concentration (436–5708 ng/mL) throughout the dosing phase with no significant differences between male and female rats. With the prolongation of the treatment time, the *in vivo* FLC exposure decreased in all groups. As Table 10 and Table 11 indicated, the AUC and C_max_ of FLC decreased and clearance increased. This discovery suggests that there was possibily an increase in xenobiotic metabolism due to the increased activity or expression of liver enzymes. Therefore, aside from the results of this study, the distribution of FLC in rat tissues, especially in the testis and epididymis, should be studied in the future to explain the mechanism of toxicity of FLC to reproductive organs, liver, brain, spleen, *etc.*

## 5. Conclusions

Histopathological and clinical pathological tests and endocrine function tests demonstrated dose-dependent testis and epididymis lesions induced by FLC. An obvious toxicity to the liver, and hematopoietic system, and potential toxicity to the brain, heart, pancreas, kidney and thymus were observed in this study. The UPLC-MS/MS method was successfully applied for the rapid and sensitive quantification of FLC in rat plasma. Toxicokinetic analysis revealed that FLC showed a persistent plasma concentration throughout the dosing phase with no significant differences between male and female rats. Nevertheless, the decreased FLC exposure *in vivo* with the prolongation of the treatment time suggested that there would be a possibility that there were gastrointestinal tract lesions affecting the absorption process with rising clearance. The causal link between FLC exposure and testis, epididymis lesion still needs further investigation.
